# Heavy Ethanol Intoxication Increases Proinflammatory Cytokines and Aggravates Hemorrhagic Shock-Induced Organ Damage in Rats

**DOI:** 10.1155/2013/121786

**Published:** 2013-09-12

**Authors:** Tsung-Ming Hu, Ru-Ping Lee, Chung-Jen Lee, Yi-Maun Subeq, Nien-Tsung Lin, Bang-Gee Hsu

**Affiliations:** ^1^Institute of Medical Sciences, Tzu Chi University, Hualien, Taiwan; ^2^Department of Psychiatry, Yuli Veterans Hospital, Hualien, Taiwan; ^3^Department of Nursing, Tzu Chi University, Hualien, Taiwan; ^4^Department of Nursing, Tzu Chi College of Technology, Hualien, Taiwan; ^5^Institute of Microbiology, Immunology and Molecular Medicine, Tzu Chi University, Hualien, Taiwan; ^6^Department of Nephrology, Tzu Chi General Hospital, Hualien, Taiwan; ^7^School of Medicine, Tzu Chi University, Hualien, Taiwan

## Abstract

Hemorrhagic shock (HS) following acute alcohol intoxication can increase proinflammatory cytokine production and induce marked immunosuppression. We investigated the effects of ethanol on physiopathology and cytokine levels following HS in acutely alcohol-intoxicated rats. Rats received an intravenous injection of 5 g/kg ethanol over 3 h followed by HS induced by withdrawal of 40% of total blood volume from a femoral arterial catheter over 30 min. Mean arterial pressure (MAP) and heart rate (HR) were monitored continuously for 48 h after the start of blood withdrawal. Biochemical parameters, including hemoglobin, ethanol, glutamic oxaloacetic transaminase (GOT), glutamic pyruvic transaminase (GPT), blood urea nitrogen (BUN), creatinine (Cre), lactic dehydrogenase (LDH), and creatine phosphokinase (CPK), were measured at 30 min before induction of HS and 0, 1, 3, 6, 9, 12, 18, 24, and 48 h after HS. Serum tumor necrosis factor-**α** (TNF-**α**) and interleukin-6 (IL-6) levels were measured at 1 and 12 h after HS. The liver, kidneys, and lungs were removed for pathology at 48 h later. HS significantly increased HR, blood GOT, GPT, BUN, Cre, LDH, CPK, TNF-**α**, and IL-6 levels and decreased hemoglobin and MAP in rats. Acute ethanol intoxication further increased serum levels of GOT, GPT, BUN, Cre, LDH, CPK, TNF-**α** and IL-6 elevation following HS. Acutely intoxicated rats exacerbated the histopathologic changes in the liver, kidneys, and lungs following HS.

## 1. Introduction

Traumatic injury is a leading cause of death and disability worldwide, and hemorrhagic shock (HS) is responsible for up to 40% of trauma deaths [1]. HS can lead to hemodynamic instability, decrease in oxygen delivery, and induce tissue hypoperfusion, leading to cellular hypoxia, organ damage, and death [2, 3]. After HS, nuclear factor-*κ*B (NF-*κ*B) is activated causing the expression of several proinflammatory cytokines, such as tumor necrosis factor-*α* (TNF-*α*) or interleukin-6 (IL-6), and this series of events can, in turn, lead to multiple organ dysfunction [2–5].

Acute alcohol intoxication is a significant risk factor for traumatic injury and causes higher morbidity or mortality rates in patients with HS [6, 7]. Acute alcohol intoxication is a clinically harmful condition that commonly follows the ingestion of a large amount of alcohol [8]. Not only risk of injury is increased by alcohol use, acute intoxication negatively affects severity of trauma-related immune compromise and recovery from trauma-related hospitalization [9]. Following HS, but also acute alcohol intoxication increases proinflammatory cytokine production and induces marked immunosuppression [6]. Hospital emergency rooms regularly see patients with HS as a result of accidents occurring while in a state of acute alcohol intoxication. This study used a rat model to explore how acute alcohol intoxication affects recovery from HS on cytokines (TNF-*α*, and IL-6) and damage to organs (liver, kidney, and lung).

## 2. Materials and Methods 

### 2.1. Preparation of Animals

Thirty-two male Wistar-Kyoto rats weighing 260–300 grams were purchased from the National Animal Center (Taipei, Taiwan). They were housed in the university Animal Center in a controlled environment at a temperature of 22 ± 1°C with a 12-hour light/dark cycle. Food and water were provided *ad libitum*. The Animal Care and Use Committee of Tzu Chi University approved the experimental protocol. 

The animals were anesthetized with ether inhalation for about 15 min. During the period of anesthesia, a polyethylene catheter (PE-50) was inserted into the femoral artery to collect blood samples and was connected to a pressure transducer (Gould Instruments, Cleveland, OH, USA) to record arterial pressure (AP) and heart rate (HR) on a polygraph recorder (Power Lab, AD Instruments, Mountain View, CA, USA). Another PE-50 catheter was inserted into the femoral vein for intravenous administration of drugs or fluid. The operation was completed within 15 min, leaving a small wound (less than 0.5 cm^2^). After the operation, the animals were placed in a conscious rat metabolic cage (Shingshieying Instruments, Hualien, Taiwan). Rats awoke soon after the operation, and acute alcohol intoxication was induced 24 h later, with the rats in a conscious state [10–12]. 

### 2.2. Acute Alcohol Intoxication

After PE-50 catheters were inserted into the femoral artery and femoral vein in rats 24 h later. Acute alcohol intoxication in rats was given 5 g/kg ethanol in normal saline mixed to 4 mL intravenously over 3 h [13].

### 2.3. Hemorrhagic Shock

HS was induced by drawing blood from the femoral arterial catheter into a 10 mL syringe after acute alcohol intoxication. An infusion pump controlled the withdrawal rate to mimic a typical bleeding event. The amount withdrawn was 40% of total blood volume (6 mL/100 gm BW + 0.77 mL) over a period of 30 min [14]. The HS procedure was followed by resuscitation with 0.5 mL normal saline at 0, 1, 3, 6, 9, 12, 18, 24, and 48 h after HS. After blood withdrawal, the animals were continuously observed for 48 h and sacrificed later for pathological study [10–12].

### 2.4. Experimental Design

**Figure 1:**
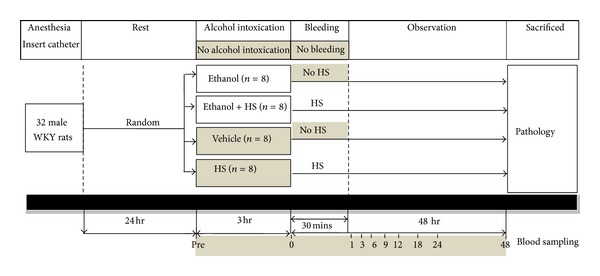
Timeline of hemorrhage and blood sampling protocols for this experiment.

Animals were randomly divided into four groups. Rats in the Ethanol group (*n* = 8), were given 5 g/kg ethanol in normal saline mixed to 4 mL intravenously over 3 h and were not subjected to HS [14]. Rats in the HS group (*n* = 8) received an intravenous drip of 4 mL normal saline for 3 h followed by induction of HS. The Ethanol + HS group (*n* = 8) were given 5 g/kg ethanol in normal saline mixed to 4 mL intravenously over 3 h after which HS was immediately induced. In the Vehicle group (*n* = 8), rats received an intravenous drip of 4 mL normal saline for 3 h and were not subjected to HS (Figure 1). 

### 2.5. Blood Sample Analysis

Arterial blood samples were obtained to determine baseline values before heparinization. Heparin (2 IU/gm BW) in 1 mL normal saline was injected via the catheter into rats over 20 min [10–12]. Arterial blood samples (0.5 mL) were collected for measurement of glutamic oxaloacetic transaminase (GOT), glutamic pyruvic transaminase (GPT), blood urea nitrogen (BUN), creatinine (Cre), lactic dehydrogenase (LDH), creatine phosphokinase (CPK), and ethanol at 3 h before induction of HS, and at 0, 1, 3, 6, 9, 12, 18, 24, and 48 h following HS, while an equal volume of 0.5 mL normal saline was used for fluid resuscitation. Blood samples of about 0.1 mL for hemoglobin (Sysmex K-1000, Sysmex American, Mundelein, IL, USA) and of 0.4 mL blood samples were immediately centrifuged at 3,000 g for 10 min. The serum was decanted and separated into two parts; one part was stored at 4°C within 1 h after collection for biochemical analysis. We measured serum levels of GOT, GPT, BUN, Cre, LDH, CPK, and ethanol with an autoanalyzer (COBAS C111, Roche Diagnostics, Basel, Switzerland) to obtain various biochemical data. The other part of the serum collected at 1 h after HS was stored at −80°C for later measurement of TNF-*α* and IL-6 concentrations [10–12]. 

### 2.6. TNF-*α* and IL-6 Measurement by ELISA

TNF-*α* and IL-6 concentrations in the blood samples were measured separately 1 and 12 hours after induction of HS by antibody enzyme-linked immunosorbent assay (ELISA) using commercial antibody pairs, recombinant standards, and a biotin-streptavidin-peroxidase detection system (Endogen, Rockford, IL, USA) as previously described [10–12]. Blood samples were collected in serum separator tubes. All reagents, samples, and working standards were brought to room temperature and prepared according to the manufacturer's directions. Reactions were quantified by optical density using an automated ELISA reader (Sunrise, Tecan Co., Grödingen, Austria) at 450/540 nm wavelengths.

### 2.7. Histological Examination

Rats were sacrificed 48 hours after induction of HS and the livers, kidneys, and lungs were immediately removed. Livers, kidneys, and lungs tissues specimens were fixed overnight in 4% buffered formaldehyde, processed using standard methods and stained with hematoxylin and eosin (H & E). One observer who was blinded to the group assignment performed the tissue analysis. The severity of liver injury observed in the tissue sections was scored as follows: 0, no evidence or minimal evidence of injury; 1, mild injury consisting of cytoplasmic vacuolation and focal nuclear pyknosis; 2, moderate to severe injury with extensive nuclear pyknosis, cytoplasmic hypereosinophilia, and loss of intercellular borders; and 3, severe necrosis with disintegration of the hepatic cords, hemorrhage, and neutrophil infiltration [10–12]. The severity of renal tubular injury was scored by estimating the percentage of tubules in the cortex or the outer medulla that showed epithelial necrosis or had luminal necrotic debris, tubular dilation, and hemorrhage: 0, none; 1, <5%; 2, 5 to 25%; 3, 25 to 75%; and 4, >75% [10–12]. Lung injury was scored as follows: 0, no evidence; 1, mild injury; 2, moderate injury; 3, severe injury with lung edema, interstitial inflammatory cell infiltration, and hemorrhage [10–12]. All evaluations were made on five fields per section and five sections per organ. 

### 2.8. Statistical Analysis

Data were expressed as mean ± SD. Statistical comparisons between different groups at corresponding time points were made by repeated measures of two-way ANOVA followed by a *post hoc *test (Bonferroni's method). Histological scores were analyzed by the Kruskal-Wallis test followed by the Dunn's test. A *P* value less than 0.05 was considered statistically significant. 

## 3. Results

### 3.1. Mean Arterial Pressure (MAP) and Heart Rate (HR)

**Figure 2:**
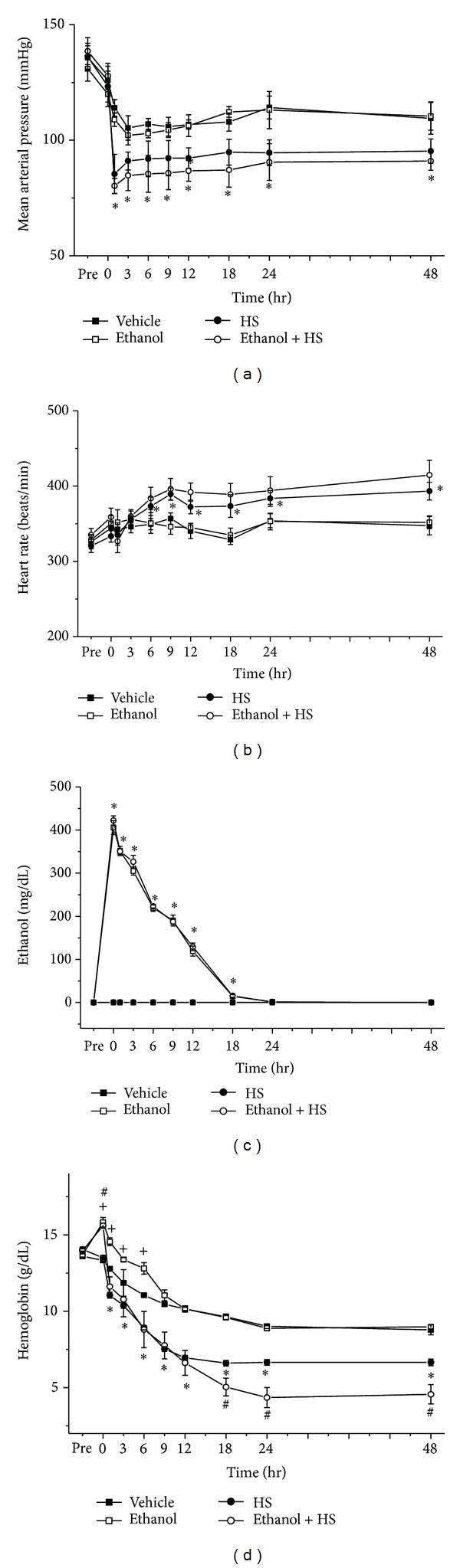
Changes in (a) mean arterial pressure, (b) heart rate, (c) serum ethanol concentration, and (d) hemoglobin following hemorrhagic shock in rats. *P < 0.05 for the HS group compared with the Vehicle group. ^+^ *P* < 0.05 for the Ethanol group compared with the Vehicle group. ^#^ *P* < 0.05 for the Ethanol + HS group compared with the HS group.

All rats were alive during the first 48 h of the study. The rats' mean arterial pressure (MAP) decreased rapidly after withdrawal of 40% of total blood volume from the femoral arterial catheter. MAP stayed relatively low during the 48 h after induction of HS (Figure 2(a)). Compared with the Vehicle group which was not subjected to HS, the Ethanol + HS group had decreased MAP at 0, 1, 3, 6, 9, 12, 18, 24, and 48 h after HS (*P < 0.05; Figure 2(a)). MAP was not significantly different between the Ethanol group and the Vehicle group (Figure 2(a)). Moreover, no significant difference was observed in MAP after HS when comparing the HS group with the Ethanol + HS group (Figure 2(a)). Heart rate (HR) was significantly increased during HS (Figure 2(b)). The HS group had increased tachycardia at 6, 9, 12, 18, 24, and 48 h after HS compared with the Vehicle group (*P < 0.05; Figure 2(b)). No significant difference in HR was observed after HS when we compared the HS group to the Ethanol + HS group (Figure 2(b)). 

### 3.2. Serum Ethanol Level and Hemoglobin

Serum ethanol levels were significantly elevated after ethanol intravenous drip in the Ethanol group and the Ethanol + HS group, with the peak at 0 h, then gradually decreasing to normal at 24 h (Figure 2(c)). Compared with the Vehicle group, the Ethanol group had higher serum ethanol levels at 0, 1, 3, 6, 9, 12, 18, and 24 h after HS (*P < 0.05; Figure 2(c)). HS did not affect serum ethanol levels when we compared the Ethanol only group with the Ethanol + HS group (Figure 2(c)). Hemoglobin gradually decreased after induction of HS (Figure 2(d)). Compared with the Vehicle group, the HS group showed decreased hemoglobin at 1, 3, 6, 9, 12, 18, 24, and 48 h after HS (*P < 0.05; Figure 2(d)), but compared with the Vehicle group, the Ethanol group had increased hemoglobin at 0, 1, 3, and 6 h after HS (^+^ *P* < 0.05; Figure 2(d)). Compared with the HS only group, the Ethanol + HS group showed increased hemoglobin at 0 h and decreased hemoglobin at 18, 24, and 48 h after HS (^#^ *P* < 0.05; Figure 2(d)). After ethanol intoxication following HS, hemoglobin increased first and then decreased compared with the HS only group.

### 3.3. Glutamic Oxaloacetic Transaminase (GOT) and Glutamic Pyruvic Transaminase (GPT)

**Figure 3:**
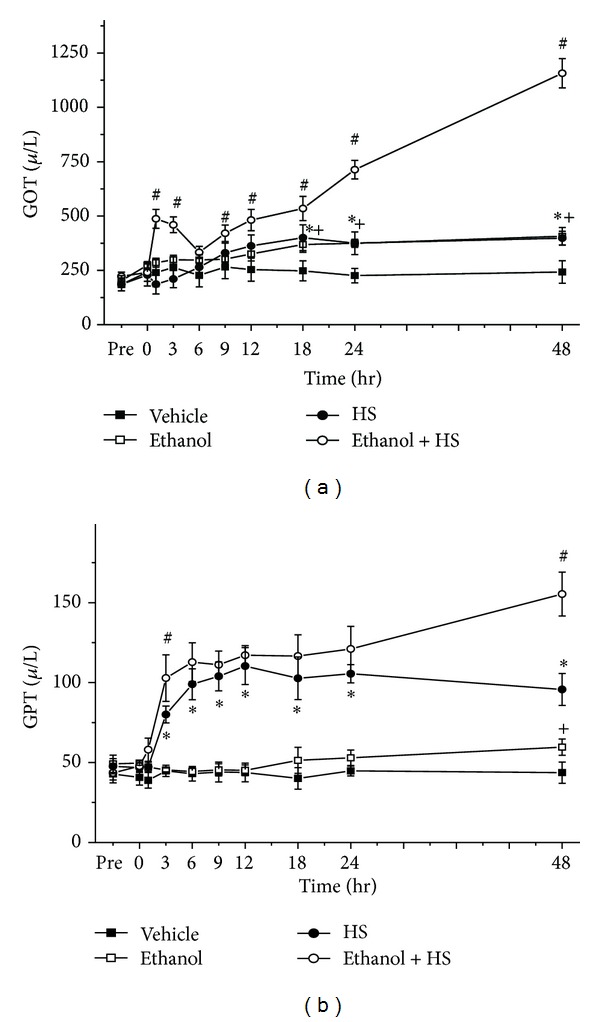
Changes in serum (a) glutamic oxaloacetic transaminase (GOT) and (b) glutamic pyruvic transaminase (GPT) after hemorrhagic shock in rats. *P < 0.05 for the HS group compared with the Vehicle group. ^+^ *P* < 0.05 for the Ethanol group compared with the Vehicle group. ^#^ *P* < 0.05 for the Ethanol + HS group compared with the HS group.

GOT and GPT are measurements of liver function. GOT gradually increased at 18, 24, and 48 h after induction of HS (*P < 0.05; Figure 3(a)). Compared with the Vehicle group, the Ethanol group showed higher GOT levels at 18, 24, and 48 h after HS (^+^ *P* < 0.05; Figure 3(a)). Compared with the HS only group, the Ethanol + HS group had higher levels of GOT at 1, 3, 9, 12, 18, 24, and 48 h (^#^ *P* < 0.05; Figure 3(a)). We observed no statistically significant difference in serum GOT in the Ethanol group compared with the HS group (Figure 3(a)). GPT gradually increased after induction of HS (*P < 0.05; Figure 3(b)). Compared with the Vehicle group, the Ethanol only group had increased GPT at 48 h after HS (^+^ *P* < 0.05; Figure 3(b)). Compared with the HS group, the Ethanol + HS group had even higher GPT at 3 and 48 h (^#^ *P* < 0.05; Figure 3(b)).

### 3.4. Blood Urea Nitrogen (BUN), and Creatinine (Cre)

**Figure 4:**
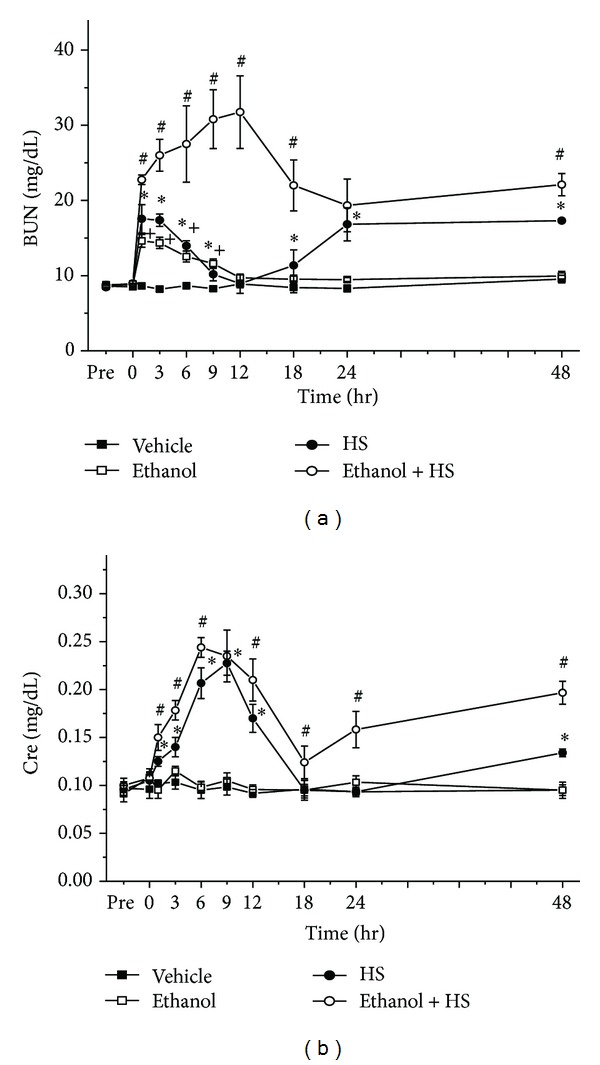
Changes in serum (a) blood urea nitrogen (BUN) and (b) creatinine (Cre) after hemorrhagic shock in rats. *P < 0.05 for the HS group compared with the Vehicle group. ^+^ *P* < 0.05 for the Ethanol group compared with the Vehicle group. ^#^ *P* < 0.05 for the Ethanol + HS group compared with the HS group.

BUN and Cre are measured of kidney function. HS increased blood BUN at 1, 3, 6, 9, 18, 24, and 48 h (*P < 0.05; Figure 4(a)). Compared with the Vehicle group, the Ethanol group had increased BUN at 1, 3, 6, and 9 h after HS (^+^ *P* < 0.05; Figure 4(a)). Compared with the HS group, the Ethanol + HS group had even higher BUN at 1, 3, 6, 9, 12, 18, and 48 h (^#^ *P* < 0.05; Figure 4(a)). No statistically significant difference was observed in serum BUN when comparing the Ethanol group with the HS group (Figure 4(a)). Serum Cre increased rapidly after induction of HS. The serum Cre values increased at 1, 3, 6, 9, 12, and 48 h after HS compared with the Vehicle group (*P < 0.05; Figure 4(b)). But compared with the HS group, the Ethanol + HS group had even higher Cre at 1, 3, 6, 12, 18, 24, and 48 h (^#^ *P* < 0.05; Figure 4(b)). 

### 3.5. Lactic Dehydrogenase (LDH) and Creatine Phosphokinase (CPK)

**Figure 5:**
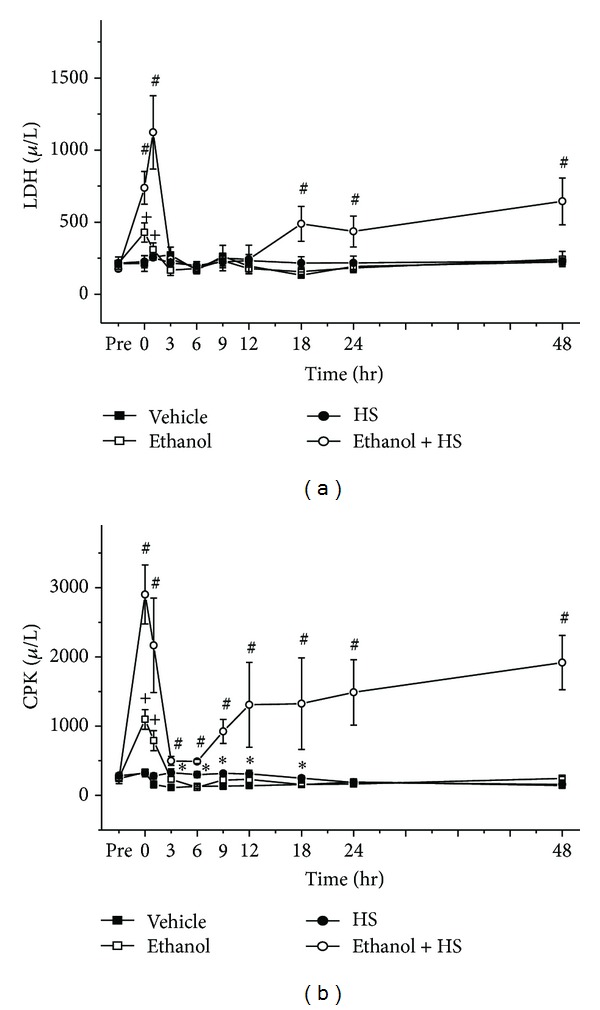
Changes in serum (a) lactic dehydrogenase (LDH), and (b) creatine phosphokinase (CPK) after hemorrhagic shock in rats. *P < 0.05 for the HS group compared with the Vehicle group. ^+^ *P* < 0.05 for the Ethanol group compared with the Vehicle group. ^#^ *P* < 0.05 for the Ethanol + HS group compared with the HS group.

The Ethanol group had increased LDH at 0 and 1 h compared with the Vehicle group (^+^ *P* < 0.05; Figure 5(a)). The Ethanol + HS group had increased LDH at 0, 1, 18, 24, and 48 h compared with the HS group (^#^ *P* < 0.05; Figure 5(a)). HS increased blood CPK at 3, 6, 9, 12, and 18 h (*P < 0.05; Figure 5(b)). Compared with the Vehicle group, the Ethanol group showed increased CPK at 0 and 1 h (^+^ *P* < 0.05; Figure 5(b)). Compared with the HS group, the Ethanol + HS group had still further increased CPK at 0, 1, 3, 6, 9, 12, 18, 24, and 48 h (^#^ *P* < 0.05; Figure 5(b)). 

### 3.6. Tumor Necrosis Factor-*α* (TNF-*α*) and Interleukin-6 (IL-6)

**Figure 6:**
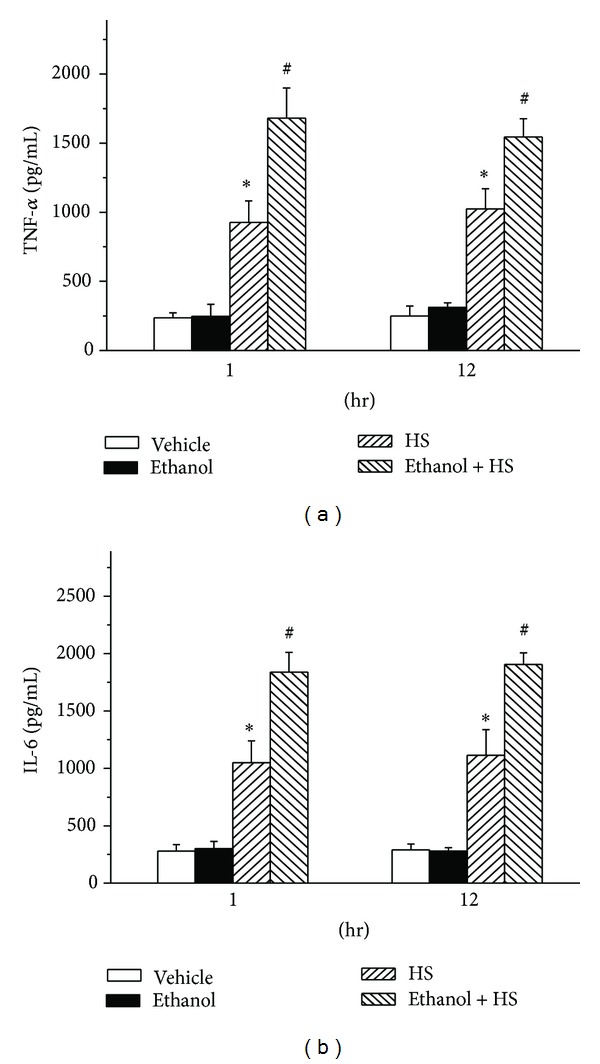
Changes in serum (a) tumor necrosis factor-*α* (TNF-*α*), and (b) interleukin-6 (IL-6) after hemorrhagic shock in rats. *P < 0.05 for the HS group compared with the Vehicle group. ^#^ *P* < 0.05 for the Ethanol + HS group compared with the HS group.

HS greatly elevated serum TNF-*α* compared with the Vehicle group at 1 and 12 h (*P < 0.05; Figure 6(a)). Prior administration of ethanol significantly increased the serum TNF-*α* at 1 and 12 h after induction of HS (^#^ *P* < 0.05; Figure 6(a)). HS increased serum IL-6, compared with the Vehicle group at 1 and 12 h after induction of HS (*P < 0.05; Figure 6(b)). Compared with the HS only group, the Ethanol + HS group had even higher IL-6 at 1 and 12 h after induction of HS (^#^ *P* < 0.05; Figure 6(b)). 

### 3.7. Histopathology of Liver, Kidney, and Lung

**Figure 7:**
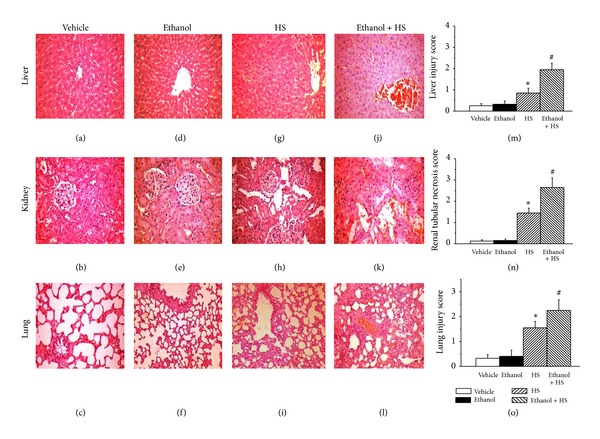
Histopathologic changes in the liver, kidneys, and lung after hemorrhagic shock in rats. Histologic sections from the Vehicle group ((a), (b), (c)), Ethanol group ((d), (e), and (f)), HS group ((g), (h), and (i)), and Ethanol + HS group ((j), (k), and (l)), stained with hematoxylin and eosin (liver, kidneys, lungs: magnification ×200). Photomicrographs (a), (d), (g), and (j) are liver sections; (b), (e), (h), and (k) are kidney sections; (c), (f), (i), and (l) are lung sections. Histopathologic injury score in liver (m), kidney (n), and lung (o) after hemorrhagic shock in rats. *P < 0.05 for the HS group compared with the Vehicle group. ^#^ *P* < 0.05 for the Ethanol + HS group compared with the HS group.

Histopathologic analysis of H & E-stained tissue sections from the liver, kidneys, and lungs after HS revealed hepatocyte necrosis and leukocytes infiltration in the liver (Figure 7(g)), tubular cell swelling, nuclear loss, tubular dilatation, and brush border loss in the kidney (Figure 7(h)). Pulmonary edema, hemorrhage, and interstitial polymorphonuclear (PMN) inflammatory cells infiltration in the lung were observed after HS (Figure 7(i)). Compared with the HS group, the Ethanol + HS group had greater histopathologic changes and hemorrhage in the liver, kidney, and lung (Figures 7(j), 7(k), and 7(l)). Compared with the HS group, the Ethanol + HS group had increased injury scores of the liver, kidney, and lung (^#^ *P* < 0.05; Figures 7(m), 7(n), and 7(o)). 

## 4. Discussion

This study found that intravenous heavy ethanol increased serum TNF-*α* and IL-6 levels after HS and aggravated HS-induced organ damage (liver, kidney, and lung) in rats.

The prevalence of alcohol-related visits to U.S. trauma centers ranged from 26.2% to 62.5% [15]. Symptoms are usually related to blood alcohol concentration. At a blood alcohol concentration higher than 300 mg/dL, there is an increased risk of respiratory depression and cardiac arrest. Death attributable to acute alcohol intoxication generally occurs at a blood alcohol concentration higher than 500 mg/dL, although the lethal dose can vary [6]. In this study, blood alcohol concentration before HS was about 405.87 ± 16.5 mg/dL—similar to concentrations observed in humans after binge drinking. 

Alcohol intoxication aggravates traumatic injury-related hemodynamic instability [16]. Low MAP at the time of arrival into the emergency department has been reported to be a predictor of poor patient outcome from traumatic injury and blood loss [17]. Alcohol intoxication may impair the ability of blunt trauma patients to compensate for acute blood loss, making them more likely to be hypotensive on admission and increasing their need for packed red blood cells and intravenous fluids [18]. Our study noted heavy ethanol intoxication after hemorrhage had lower MAP and tachycardia than ethanol group. But there was no significant difference in MAP and HR between the HS group and the Ethanol + HS group in this study. We observed that after ethanol intoxication, hemoglobin increased first, which may be due to the higher osmolality of ethanol induced hemoconcentration. However, hemoglobin decreased at 18, 24, and 48 h following hemorrhage in acute alcohol intoxicated rats compared with the HS only group. This might be because acute alcohol intoxication affected tissue hemorrhage following HS. Pathology examination proved that the Ethanol + HS group had more tissue hemorrhage in the liver, kidney and lung. 

The liver is particularly at risk for alcohol-related damage because it receives portal blood directly from the intestinal tract and thus experiences the highest concentration of alcohol presented to any organ [19]. In addition, ethanol metabolism in the liver produces potentially harmful toxic metabolites such as acetaldehyde, acetate, and reactive oxygen species [20]. Increased serum TNF-*α* and IL-6 concentrations have frequently been found in alcoholic liver cirrhosis patients [21]. Alcohol intoxicated rodents, present with lower blood pressure at the time of injury, have decreased tolerance to blood loss and have impaired blood pressure recovery during fluid resuscitation. The accentuated hypotension leads to tissue hypoperfusion, which enhances susceptibility to tissue injury reflected in greater elevation in circulating liver function [22]. Our study also observed that acute ethanol intoxication aggravated liver damage by greater elevation of GOT, GPT, and the histopathologic analysis of liver revealed more hepatocyte necrosis and leukocytes infiltration following hemorrhage in acute alcohol intoxicated rats. 

Alcohol consumption increased malondialdehyde levels, superoxide dismutase, and catalase activity significantly in alcohol intoxicated rats [23]. Alcohol intoxicated rats result in a greater reduction of blood flow to the kidney than that seen in nonintoxicated rats after HS [24]. The accentuated hypotension leads to tissue hypoperfusion, which enhances susceptibility to tissue injury reflected in greater elevation in circulating renal function [23]. Our study found that acute ethanol intoxication induced greater renal damage after HS in rats by elevated serum BUN, Cre and exacerbated the histopathologic changes in kidneys following HS in rats. 

Alcohol exposure of the host can predispose to pneumonia infection [9]. Acute alcohol intoxication exacerbates the HS-induced increase in lung proinflammatory cytokine TNF-*α* expression in rats [25]. In other studies, alcohol-treated mice had worse clinical outcomes, deteriorated pulmonary structure, and increased levels of IL-6 compared with the nonalcohol treated mice [26]. Our study found that heavy ethanol intoxication aggravated lung damage including pulmonary edema, hemorrhage, and interstitial PMN inflammatory cell infiltration in the lung following hemorrhage. 

In response to HS, NF-*κ*B is involved in apoptosis and the inflammatory cascade [5]. The organism provokes release of proinflammatory cytokines (TNF-*α* and IL-6) into surrounding tissues, thereby causing tissue damage and organ failure [2, 3]. TNF-*α* and IL-6 peak early after HS and continue elevating during HS because tissue hypoperfusion persist [10–12]. Treatment with anti- TNF-*α* antibodies reduced organ injury and improved survival in rats after HS [27]. Inhibition of the synthesis of IL-6 may exert beneficial effects on HS [28]. Acute alcohol intoxication can increase proinflammatory cytokine production and induce marked immunosuppression after HS [6]. Our study found that intravenous injection of heavy ethanol increased serum TNF-*α* and IL-6 production after HS in rats. Altered inflammatory cell and adaptive immune responses after alcohol consumption result in increased of infections and other organ-specific immune-mediated effects [9]. 

Sex differences in alcohol drinking is somewhat equivocal in rodent studies: one study noted female rodents tend to drink more alcohol than males [29] and other study noted adolescent males have been reported to drink more than females [30], whereas others suggest that there is no sex difference [31, 32]. Increases in pubertal hormones, including gonadal and stress hormones, are a prominent developmental feature of adolescence and could contribute to the progression of sex differences in alcohol drinking patterns during puberty [33]. Our study used adult male Wistar-Kyoto rats and found increased serum TNF-*α* and IL-6 levels after HS and aggravated HS-induced organ damage. Further studies are required to investigate sex differences in alcohol drinking behavior and/or the influence of pubertal hormone changes on the effects of ethanol on physiopathology and cytokine levels following HS in acutely alcohol-intoxicated rats.

Recent study noted that rats were given a single oral dose of ethanol (5 g/kg, 30%) increased survival after HS and decreased HS-induced liver injury [34]. However, the blood ethanol concentration in this study is unknown and only given a single oral dose of ethanol. Our study, blood alcohol concentration before HS was similar to concentrations observed in humans after binge drinking. Acute ethanol intoxication leads to a dysregulation of the hemodynamic, neuroendocrine, inflammatory, and immune responses to hemorrhage. This disruption of the normal neuroendocrine counterregulatory response impairs hemodynamic stability and recovery, contributing to compromised tissue perfusion and increased end-organ injury [6, 22]. Intravenous heavy ethanol injection of this study also noted increased serum TNF-*α* and IL-6 levels after HS and aggravated HS-induced organ damage in rats.

## 5. Conclusion

Acute ethanol intoxication increased serum TNF-*α* and IL-6 levels following HS, along with aggravating HS-induced organ damage in rats.
